# Global statements to produce and implement evidence in the post-COVID-19 era provide a path forward for rehabilitation - A joint initiative of Cochrane Rehabilitation and the leading journals in the field

**DOI:** 10.1007/s10926-022-10071-6

**Published:** 2022-10-07

**Authors:** Stefano Negrini, Kristian Borg, Anne Cusick, Giorgio Ferriero, Walter R. Frontera, Douglas P. Gross, Allen Heinemann, Wendy Machalicek, Ann Patricia Moore, Randolph J. Nudo, Dominic Pérennou, Henk Stam, Carlotte Kiekens

**Affiliations:** 1 Department of Biomedical, Surgical and Dental Sciences, University “La Statale”, Milan, Italy; 2 IRCCS Istituto Ortopedico Galeazzi, Milan, Italy; 3 Department of Clinical Sciences, Karolinska Institutet Danderyd University Hospital, Stockholm, Sweden; 4 Discipline of Occupational Therapy, Faculty of Medicine and Health, The University of Sydney, Sydney, Australia; 5 Physical and Rehabilitation Medicine Unit, Scientific Institute of Tradate, IRCCS, Istituti Clinici Scientifici Maugeri, Tradate, Italy; 6 Department of Biotechnology and Life Sciences, University of Insubria, Varese, Italy; 7 Department of Physical Medicine, Rehabilitation, and Sports Medicine and Department of Physiology, University of Puerto Rico School of Medicine, San Juan, Puerto Rico; 8 Department of Physical Therapy, University of Alberta, Edmonton, AB Canada; 9 Department of Physical Medicine and Rehabilitation, Feinberg School of Medicine, Northwestern University, Chicago, IL USA; 10 Center for Rehabilitation Outcomes Research, Shirley Ryan AbilityLab, Chicago, IL USA; 11 Department of Special Education and Clinical Sciences, College of Education, University of Oregon, Eugene, OR USA; 12 Professor Emerita, University of Brighton, East Sussex, UK; 13 Department of Rehabilitation Medicine, University of Kansas Medical Center, Kansas City, KS USA; 14 Department of NeuroRehabilitation South Hospital, Université Grenoble Alpes, Grenoble, France; 15 Erasmus University Medical Center, Rotterdam, The Netherlands; 16 IRCCS MultiMedica, Milan, Italy

Three fundamental resources to promote and support evidence were published at the end of 2021 and the start of 2022. The purpose of these contributions was to emphasize one of the main lessons learned from the COVID-19 pandemic and specifically its impact on medicine: the importance of using evidence to make decisions. These initiatives captured the attention of *Nature* [1], with an editorial that focused on the impact that evidence could and should have beyond health, informing decisions relevant to global challenges, using the best available up-to-date or “living” evidence. The *Nature* editorial pointed out the low quality of many publications dedicated to COVID-19 during the pandemic, an opinion shared by editors of rehabilitation journals, who also noticed an increase in the incidents of misconduct, in particular attempts of duplicate publications. In this paper, we summarize for the rehabilitation audience the main recommendations of the 3 groups that worked simultaneously but independently on the use of evidence in health decision-making. The conclusions were similar, a finding that reinforces their importance.

The World Health Organization (WHO) Evidence-informed Policy Network (EVIPNet) published the document *“Together on the road to evidence-informed decision-making for health in the post-pandemic era: a call for action”* [2]. The document recommends 4 main actions (Table 1), mainly directed to governments and policy decision-makers: 1) institutionalize structures and processes to support evidence-informed decision-making; 2) use high-quality norms, standards and tools promoting evidence-informed decision-making; 3) strive to ensure national and international capacity for the translation and use of evidence in decision-making; and 4) strive to ensure that evidence is accessible, timely and relevant for policymaking, especially in emergencies. Each action is supported by enabling strategies that provide a practical way forward for implementation. As stakeholders in health and social systems and as part of the evidence ecosystem, readers can promote, support and implement these actions.

**Table 1:** Recommendations by the World Health Organization Evidence-informed Policy Network (EVIPNet) in the document “*Together on the road to evidence-informed decision-making for health in the post-pandemic era: a call for action*”

**1. Institutionalize structures and processes to support evidence-informed decision-making**	
We call on governments and intergovernmental organizations:	
1.1	to assess, create and strengthen institutional structures and processes that are agile and can rapidly respond to decision-makers’ needs, while drawing upon a range of types of evidence that are contextualized and actionable to inform decision-making;
**1.2**	to ensure that these structures and processes are (i) demand-driven, (ii) ethical, (iii) multisectoral and multidisciplinary in nature, (iv) adapted to the local context, and (v) positioned to coordinate their resources effectively to avoid duplication of evidence production;
**1.3**	to support and advocate for routine and transparent evidence-to-policy co-production processes that are equity-oriented, inclusive and foster multisectoral participation of all stakeholders, including systematic approaches to elicit input and engage citizens to encourage democratic legitimacy, accountability and transparent governance;
**1.4**	to demonstrate leadership and commitment by taking action, such as adopting a formal resolution, to accelerate, advance and institutionalize evidence-informed decision making at the global, regional and national levels to better prepare for and confront future health emergencies as well as routine societal challenges;
**1.5**	to promote a climate of and build a culture for evidence-informed decision-making so that individual stakeholders, institutions and societies as a whole value, understand and routinely use evidence;
**1.6**	to strengthen monitoring and evaluation of evidence-informed decision-making processes, including impact assessment of measures, to enhance the knowledge base of evidence-to-policy activities and their institutionalization, improve interventions, and reinforce accountability and learning
**2. Use high-quality norms, standards and tools promoting evidence-informed decision-making**	
We strongly encourage:	
2.1	intergovernmental organizations to develop, provide access to and disseminate agreed upon norms, standards and tools for evidence-informed decision-making, ensuring rigorous, transparent and systematic processes, including the development of a tool that describes key characteristics of national institutional structures and processes;
2.2	intergovernmental organizations to collect, disseminate and support the scaling up of good practices and lessons learned on national, regional and international evidence informed decision-making activities, and to provide opportunities for peer support and learning;
2.3	governments and intergovernmental organizations to collaborate to adhere to standards for evidence-informed decision-making
**3. Strive to ensure national and international capacity for the translation and use of evidence in decision-making**	
We stress the importance for:	
3.1	intergovernmental organizations to provide technical support and assistance to Member States to strengthen their national institutional capacity, structures and processes to support evidence-informed decision-making processes in a timely and responsive manner, and build trust and legitimacy around evidence-informed decision making;
3.2	governments to ensure that a critical mass of people is trained across the evidence spectrum and in using evidence to formulate policies, e.g. by promoting the inclusion of evidence-informed decision-making courses in the curricula of universities and other regular training programmes;
3.3	governments and intergovernmental organizations to increase synergies and systemic capacities by strengthening collaboration across the evidence ecosystem and moving away from siloed approaches, to coordinate and integrate research, data and expertise across stakeholders and sectors in transparent ways for more effective and timely decision-making;
3.4	governments and intergovernmental organizations to secure sustainable funding and incentives for evidence-informed decision-making activities
**4. Strive to ensure that evidence is accessible, timely and relevant for policy-making, especially in emergency situations**	
We invite:	
4.1	intergovernmental organizations to develop and provide global public goods, such as relevant, timely and high-quality global evidence syntheses and guidelines that are easily adaptable to and can be used at local levels;
4.2	intergovernmental organizations and other international, regional and national stakeholders to establish and maintain comprehensive evidence repositories to provide easy and affordable access for countries of all income levels;
4.3	governments and intergovernmental organizations to advocate for “Open Science”, a movement to make scientific research accessible, and to ensure that policy-makers have easy access to contextualized sources of evidence for health

The COVID-19 Evidence Network to support Decision-making (COVID-END) [3] is a global organization launched by McMaster University in Canada at the start of the pandemic to cope with COVID-19 by using the best available evidence. COVID-END includes most organizations active in the prevention and management of COVID-19, including Cochrane [4] and Cochrane Rehabilitation [5]. In 2021, COVID-END convened the Global Commission on Evidence to Address Societal Challenges to change the global panorama on evidence generation beginning with the lessons learned during the COVID-19 pandemic. The commission published a report titled “*A wake-up call and path forward for decision-makers, evidence intermediaries, and impact-oriented evidence producers*” [6]. The title flags the need for immediate, targeted action to ensure high-quality, timely, relevant and feasible decision-making in systems affecting individual, family, community and societal wellbeing. Core to the report is the concept of the best available research evidence. The report preamble explains “*now is the time… [for] creating the capacities, opportunities and motivation to use evidence to address societal challenge, and putting in place the structures and processes to sustain them*”. The commission explored the levels, sectors and complexity of societal challenges needing evidence; decision-making processes and who decision-makers are; forms of evidence encountered in decision-making; how forms of evidence can be mapped to decisions; the need for high-quality local and global evidence; the critical role of system infrastructure for evidence-based decision-making; and the role of evidence intermediaries, public goods and distributed capacity. The report presents recommendations that encompass the framing/approach, structures and processes, accountabilities and funding, together with actions that emerge from these foundations. The document includes 8 main and 24 total recommendations clearly presented in short-form in the executive summary of the report (Table 2). As stakeholders in health in roles that encompass decision-makers, evidence intermediaries and evidence producers, readers may appreciate this report recommendation to all stakeholders: “*Citizens should consider making decisions about their and their families’ well-being based on best evidence; spending their money on products and services that are backed by best evidence; volunteering their time and donating money to initiatives that use evidence to make decisions about what they do and how they do it; and supporting politicians who commit to using best evidence to address societal challenges and who commit (along with others) to supporting the use of evidence in everyday life*” [6].

**Table 2:** Recommendations by the Global Commission on Evidence promoted by the COVID-19 Evidence Network to support Decision-making (COVID-END). In bold are the 8 main recommendations

All decision-makers, evidence intermediaries and impact-oriented evidence producers	
**1**	**Decision-makers, evidence intermediaries and impact-oriented evidence producers should recognize the scale and nature of the problem**
**2**	All decision-makers should pay attention when a claim is being made and ask about the quality and applicability of the evidence on which the claim is based
Multilateral organizations	
**3**	**The UN, the G20 and other multilateral organizations should endorse a resolution that commits these multilateral organizations and their member states to broaden their conception of evidence, and to support evidence-related global public goods and equitably distributed capacities to produce, share and use evidence**
**4**	**The World Bank should dedicate an upcoming World Development Report to providing the design of the evidence architecture needed globally, regionally and nationally, including the required investments in evidence related global public goods and in equitably distributed capacities to produce, share and use evidence**
Government policymakers	
**5**	**Every national (and sub-national) government should review their existing evidence-support system (and broader evidence infrastructure), fill the gaps both internally and through partnerships, and report publicly on their progress**
**6**	Government policymakers should ensure that the executive and legislative branches of government have access to the staff, partnerships and other resources needed for evidence support
**7**	Government policymakers should select their science advisors based on their ability to find, contextualize and communicate diverse forms of evidence, and to sustain a high-performing evidence-support system
**8**	Government policymakers should hold advisory bodies to higher standards in their use of evidence
**9**	Government policymakers should complement their general support for data collection and sharing with specific support for a more diversified evidence base that can inform decision-making in equity sensitive ways
**10**	Government policymakers should incentivize open science as a key enabler for using evidence in decision-making
**11**	Government policymakers should ensure that regulatory regimes and ongoing validation schemes for artificial intelligence (AI) optimize AI’s benefits for evidence-support systems and minimize its harms
Organizational leaders, professionals and citizens	
**12**	Every significant organizational association, professional body and impact-oriented civil-society group should review its contributions to its national (or subnational) evidence-support system (and broader evidence infrastructure), fill the gaps both internally and through partnerships, and report to its members on their progress
**13**	**Citizens should consider making decisions about their and their families’ well-being based on best evidence; spending their money on products and services that are backed by best evidence; volunteering their time and donating money to initiatives that use evidence to make decisions about what they do and how they do it; and supporting politicians who commit to using best evidence to address societal challenges and who commit (along with others) to supporting the use of evidence in everyday life**
Evidence intermediaries	
**14**	**Dedicated evidence intermediaries should step forward to fill gaps left by government, provide continuity if staff turn-over in government is frequent, and leverage strong connections to global networks**
**15**	**News and social-media platforms should build relationships with dedicated evidence intermediaries who can help leverage sources of best evidence, and with evidence producers who can help communicate evidence effectively, as well as ensure their algorithms present best evidence and combat misinformation**
**16**	All evidence intermediaries should – in a timely and responsive way – support the use of best evidence to answer the question being asked (or that should be asked given the decision-maker’s area of interest)
Impact-oriented evidence producers	
**17**	Evidence groups should anticipate and fill gaps in, and adhere to standards for, their respective forms of evidence
**18**	Evidence groups should play to their comparative advantages, collaborate with groups that have complementary comparative advantages, and help to build a better evidence-support system in their country and a better global evidence architecture
**19**	Evidence groups should be open to adapting innovations from other sectors
**20**	Evidence groups should ensure they have the agility to pivot to new topics when global emergencies strike
**21**	Evidence groups should prepare ‘derivative products’ that communicate what we know (and with what certainty we know it) in ways that make sense to their target audiences
**22**	Academic institutions, and their public funders, should incentivize faculty members to contribute to their national (or sub-national) evidence-support system and to evidence-related global public goods
**23**	Journal publishers should improve the ways in which they support the use of best evidence
Funders	
**24**	**Governments, foundations and other funders should spend ‘smarter,’ and ideally more, on evidence support**

Finally, Cochrane [4] published “Cochrane Convenes: Preparing for and responding to global health emergencies. Learnings from the COVID-19 evidence response and recommendations for the future” [7]. This incisive and extensive work captures 3 over-arching reflections that should jolt us all to action: the pandemic-exacerbated pre-existing inequities in society, including social determinants of health, and the evidence response has been globally unequal; the rapidly changing context and rapidly evolving evidence of mixed quality led to particularly challenging communication of the certain and uncertain; and strategies to prevent or disarm misinformation and disinformation were ineffective or insufficient. Three areas for action arise from these lessons learned: the need to incentivise and encourage change at the system level; produce and share research and evidence synthesis; and reflect on communicating uncertainty as well as understand misinformation/disinformation and do something about it (Table 3). Each area has specific strategies that can be implemented by stakeholders. Although the document is more specific about the evidence production and dissemination process, it also takes into account policymaking.

**Table 3:** Key recommendations from “*Cochrane Convenes (2022): Preparing for and responding to global health emergencies: Learnings from the COVID-19 evidence response and recommendations for the future.*”

**Incentivizing and encouraging change at system level**	
	At system level, in order to prepare to serve the needs of decision makers equitably and with high-quality evidence during the next global health emergency, Cochrane Convenes participants recommend:
	providing more financial support for evidence generation, communication, networks and infrastructure in low- and middle-income countries
	working with national and international stakeholders to describe the ideal global evidence system, or service, and what this might require – and then advocating for the necessary conditions
	working towards greater transparency about how (and what) evidence is used in decision making
	harnessing research commissioning and financing as tools to help identify, prioritize, fund and meet national and international research needs equitably
**Reviewing the way research and evidence syntheses are produced and shared**	
	At a research and research institution level, Cochrane Convenes participants recommend:
	further developing or reviewing research tools, processes, methods and standards to meet the challenges of rapid onset global health emergencies more effectively
	investing in and using new technology to facilitate review processes (using study repositories and databases, crowd screening, and artificial intelligence) and enhance transparency and data sharing
	evaluating the suitability of faster, more agile editorial processes and formats (rapid/ living reviews and preprints)
	investing time and resources in science communications on an ongoing basis – including in people, technology and learning, as well as evaluating what works
	Other recommendations highlight the value of being good partners in support of the changes and recommendations made at system and communication levels, including:
	being alert to – and communicating about – fraudulent trials and studies
	reducing duplication and research waste
	playing a role in building capacity in low- and middle-income countries
	engaging with evidence users – directly and in partnership with others – to help communicate uncertainty and the evolving nature of the evidence
**Reflecting on uncertainty, misinformation and disinformation**	
	Top-line recommendations on what is needed include:
	researching what works (and where) in terms of both communicating uncertainty and countering mis/disinformation
	building trust through increased collaboration between evidence producers, evidence users and clinical partners
	increasing transparency around public decision-making processes
	considering a form of accreditation and quality approval for official sources of evidence that has met certain quality-control standards making it easier for people to access trustworthy information – considering, for example, the increased engagement of information scientists to help increase both ‘push’ (ensuring people receive and can act on evidence) and ‘pull’ (helping people to find and use evidence), as well as using non traditional formats, channels and champions
	forming multidisciplinary coalitions to hold those deliberately creating and sharing mis/disinformation to account

Evidence-based medicine (EBM) and evidence-based practice in health are only a few decades old and combine the 3 components of research-based evidence: the clinician's expertise and the patient's values and preferences [8]. An essential role of EBM is to strengthen the importance of scientific data in decision-making in medicine, which is increasingly complex given the exponential growth of research and information. How do we identify the best available information? How do we make decisions about the care of individual patients and populations? These are some of the fundamental questions that EBM answers. In this paper, we focus on the first component of the triad: research-based evidence. For all professionals working in health care, EBM makes the basic assertion that we cannot provide quality patient care without evidence. EBM is, arguably, the best way forward for medicine. The importance of evidence is also noted, for example, in the social sciences with the Campbell Collaboration, the social science research network. The documents mentioned above emphasize the need to extend and establish the use of evidence in the process of making policy decisions, particularly, but not limited to, health policy.

EBM in rehabilitation has not always been accepted as the best way forward [9]. Rehabilitation focuses on functioning and is based on conceptual models that are close to the complex bio-psycho-social paradigm. Evidence gathering is complicated, and the conduct of a classical randomized controlled trial (RCT), the gold standard study design for generating evidence in many areas of medicine, may be challenging and in fact unfeasible for many questions in rehabilitation science. Indeed, the RCT is less appropriate when complex interventions and multiple interactions are studied [10]. Additionally, heterogeneity in patient populations can pose difficulties in obtaining a sufficiently powered sample size for an RCT, and recruitment to traditional no-treatment control conditions can be challenging and present ethical concerns. A narrow approach to evidence, based on only RCTs and confusion between the means and the aim, has contributed significantly to the diffidence in rehabilitation science to accept EBM. Other reasons include challenging methodological research issues in our field [11] and the difficulties associated with the reporting of results [12]. Nevertheless, it has become clear that the practice of rehabilitation benefits from and is in need of an EBM approach.

The documents highlighted in this paper call for evidence as the main tool to make decisions about the treatment of health conditions in individuals and populations. This approach to decision-making is becoming clearer to policymakers, too. The documents call us to action or provide the resources to support action for evidence-based decision making in health and in societal challenges that face us locally and globally. We are stakeholders in health and human systems and in the evidence-ecology that have the opportunity to feed into decision-making systems that affect us all. We have to enhance evidence-informed decision-making in our own practice and in the systems in which we live and work. We can be decision-makers or decision intermediaries, adopting or advocating for the specific strategies outlined in sources presented here in practice, policy and education. We can be evidence-producers, advancing the strength and quality of research by asking questions suited to answers that use well-designed randomised controlled designs, primarily because these provide the greatest opportunity for synthesis and uptake in clinical guidelines. When other questions are asked and other research designs are used, we can build capacity to ensure the appropriate interpretation and application of less rigorous findings. Beyond intervention research, rehabilitation systems and services need high-quality evidence to inform the managerial and administrative decision-makers who ultimately control access to and provision of human and infrastructure resources.

The world of rehabilitation cannot afford to do without evidence, nor to remain diffident and passive on this issue. Our campaign to improve evidence in rehabilitation is fundamental to the future of the field, and we need to identify the optimal approach to the generation and utilization of evidence appropriate for rehabilitation. First, wherever appropriate and possible, we need to conduct well-designed RCTs. When RCTs are not appropriate or possible, other types of study designs such as rigorous quasi-experimental and n-of-1 designs can be used depending on the nature of the research question. Second, the 3 documents summarized in this editorial repeatedly stress the need for collaboration. To implement the many strategies and work toward achievement of the many recommendations, we need to champion rehabilitation as an essential, multidisciplinary, collaborative field. In rehabilitation, collaboration is particularly required, and divisions of any type (cultural, professional, and other) interfere with efforts to generate the best evidence and strengthen the field. This emphasis on collaboration includes other fields of inquiry because it facilitates a supportive environment, with evidence guiding discussions and decisions beyond healthcare systems to health policy and other areas important to society.

Rehabilitation as a field and the community of rehabilitation journals are not new to collaborations. Rehabilitation journals have co-published several important papers during the last decade on various topics, including implementation of reporting guidelines [13] and trial repositories [14], the WHO “Rehabilitation 2030: a call for action” [15] and specific relevant research initiatives [12]. Cochrane Rehabilitation and rehabilitation journals are committed to “collaborate [as] groups that have complementary comparative advantages, and help to build a better evidence-support system … and architecture”, “improve the ways in which [we] support the use of best evidence” and to “prepare derivate products communicating what we know in ways that make sense to their target audiences” [5]. Finally, we are also committed to “investing time and resources in science communications on an ongoing basis”, “being alert to – and communicating about – fraudulent trials and studies”, “reducing duplication and research waste”, and “engaging with evidence users to help communicate uncertainty and the evolving nature of the evidence” [6].

This paper is supported and co-published by the following journals, and their Editors in Chief:

- Annals of Physical and Rehabilitation Medicine – Dominic Pérennou
- American Journal of Physical Medicine & Rehabilitation – Walter Frontera
- Developmental Neurorehabilitation – Wendy Machalicek
- European Journal of Physical and Rehabilitation Medicine – Stefano Negrini and Giorgio Ferriero
- Journal of Occupational Rehabilitation – Douglas Gross
- Journal of Rehabilitation Medicine – Kristian Borg and Henk Stam
- Musculoskeletal Science & Practice – Ann Moore
- Neurorehabilitation and Neural Repair – Randolph Nudo
